# Gnatophyma associated with Morbihan disease: An unusual form of rosacea successfully treated with ablative carbon dioxide laser

**DOI:** 10.1111/jocd.16435

**Published:** 2024-06-23

**Authors:** Amal Chamli, Houda Hammami, Malek Mrad, Refka Frioui, Anissa Zaouak, Samy Fenniche

**Affiliations:** ^1^ Dermatology Department Habib Thameur Hospital Tunis Tunisia; ^2^ University of Tunis El Manar, Faculty of Medicine of Tunis Tunis Tunisia

Dear Editor,

A 45‐year‐old man of IV Fitzpatrick skin type, presented to our dermatology department with facial redness and swelling associated with a gradual enlargement of the chin over the past 8 months, resulting in a compromised cosmetic appearance. Dermatological examination revealed a well‐defined, infiltrated erythematous and edematous plaque measuring 5 cm×3 cm on the chin, with the coalescence of multiple skin‐colored nodules on the surface. The skin exhibited thickening with dilated pores, and the remainder of the face displayed erythematous edema (Figure 1). Otherwise, the patient was asymptomatic, reporting no pruritus, pain, or flushing. A skin biopsy was done and showed a dermis rich in pilosebaceous follicles with dilated capillaries surrounded by a dense polymorphous inflammatory infiltrate. Based on clinical and pathological data, the diagnosis of Morbihan syndrome was made. He was treated with doxycycline and topical metronidazole with slight improvement. The patient was not satisfied, and his chief complaint was the enlargement of the chin. Considering the cosmetic concerns, treatment with carbon dioxide (CO_2_) ablation was proposed for the patient. Local anesthesia using 1% lidocaine and a 1:100 dilution of epinephrine solution was administered throughout the entire affected area. Hence, an excision of hypertrophied tissue was done using a continuous CO_2_ laser (10 600 nm, Limmer Laser, Germany). Two distinct techniques were employed: a continuous focused beam (20 W) for nodule excision and a defocused beam (20 W) for gradual layer‐by‐layer vaporization of hypertrophied tissue, facilitating chin contour reshaping (Figure 2).

**FIGURE 1:**
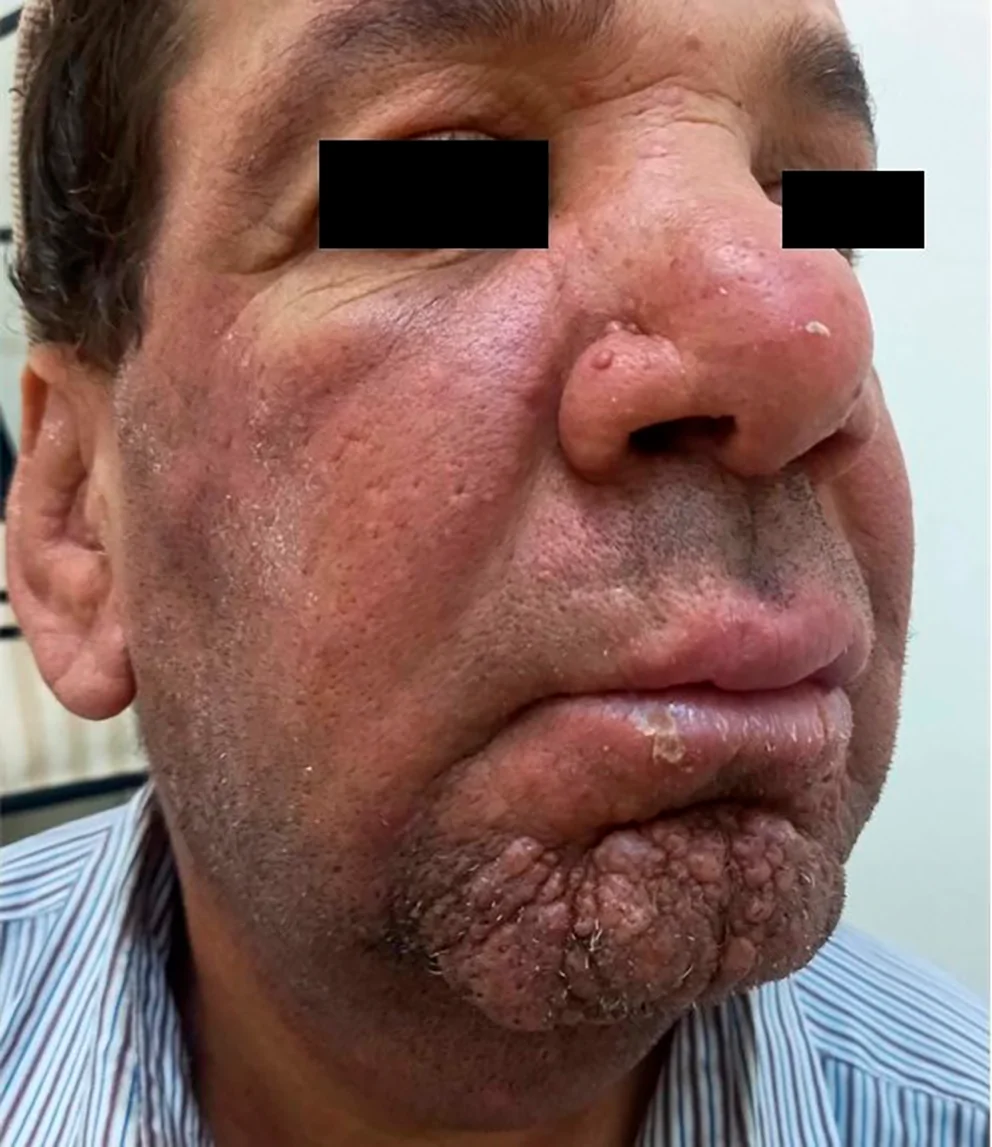
Clinical aspect at baseline shows an erythematous infiltrated and edematous plaques over the face and is more marked in the lower part of the face, associated with multiple nodules and an enlargement of the chin.

**FIGURE 2:**
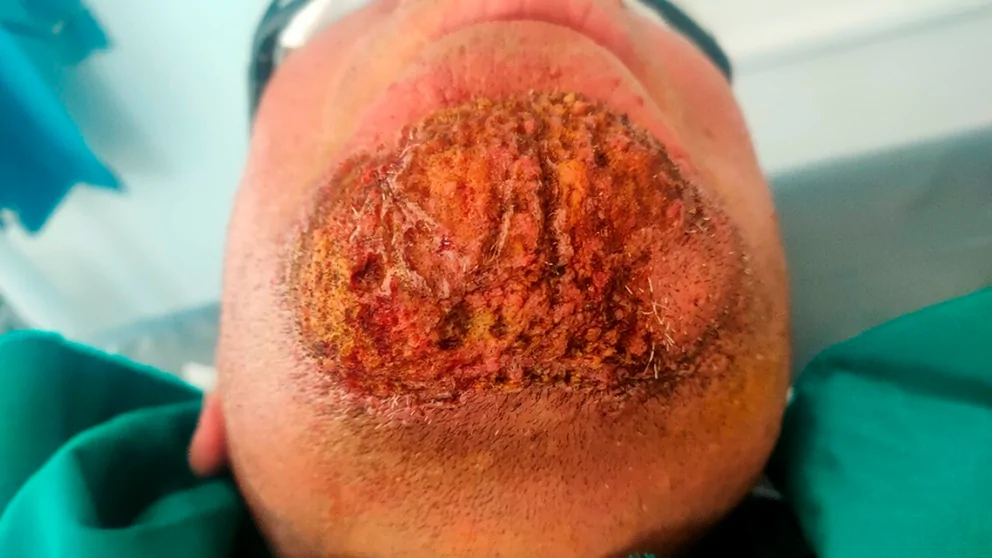
Immediately after the first session of CO_2_ laser ablation.

Initially, a focal technique was employed for the excision of nodular lesions using continuous cutting in focused mode. The papules were secured with toothed forceps on the top and gently pulled to the side over wet gauze to prevent charring of the surrounding normal skin, and the laser was directed at the base of the papule. Subsequently, a vaporization technique (defocused) was applied using a freehand defocused continuous‐mode CO_2_ laser across the entire anatomical area of the chin with gentle circular movements, progressively expanding the ablated area to optimize aesthetic outcomes and homogenize the treated region. Tissue was excised in successive layers in several passes. Following each pass, epidermal debris was cleared using a non‐woven compress soaked in physiological saline. The wound was meticulously dried after each cleaning episode. Forced air cooling was not employed.

Postoperative wound care involved washing the wound and applying Vaseline multiple times daily to soften the crusts, accompanied by using a reparative cream to facilitate lesion healing. The outcome was assessed 2 weeks after the laser sessions. The re‐epithelialization of the treated surfaces was achieved in a remarkably short time, with no reported side effects. Aesthetic improvements were evident, characterized by reductions in hypertrophy, enhancements in skin texture, and a diminishment of papules (Figure 3). However, a few persistent surface irregularities persisted. Subsequently, a second session was performed using a vaporizing technique employing a defocused beam at a lower power setting (10 W) to further refine aesthetic outcomes. Significant enhancement was achieved, resulting in a more symmetrical and more defined chin devoid of scarring. The patient expressed profound satisfaction with the ultimate cosmetic outcome. The patient was seen after 2 months, and the results were maintained (Figure 4).

**FIGURE 3:**
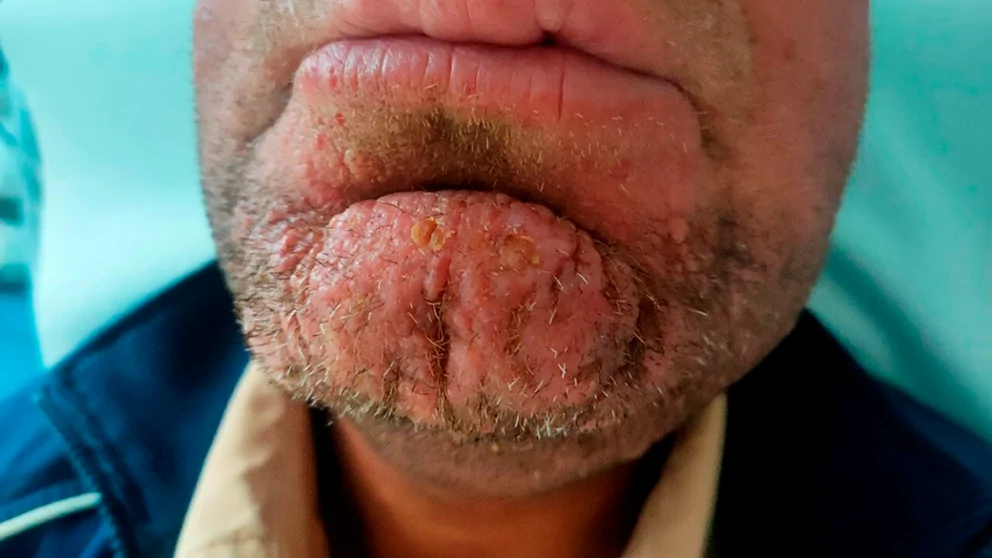
Result 2 weeks after the first session of CO_2_ laser treatment.

**FIGURE 4:**
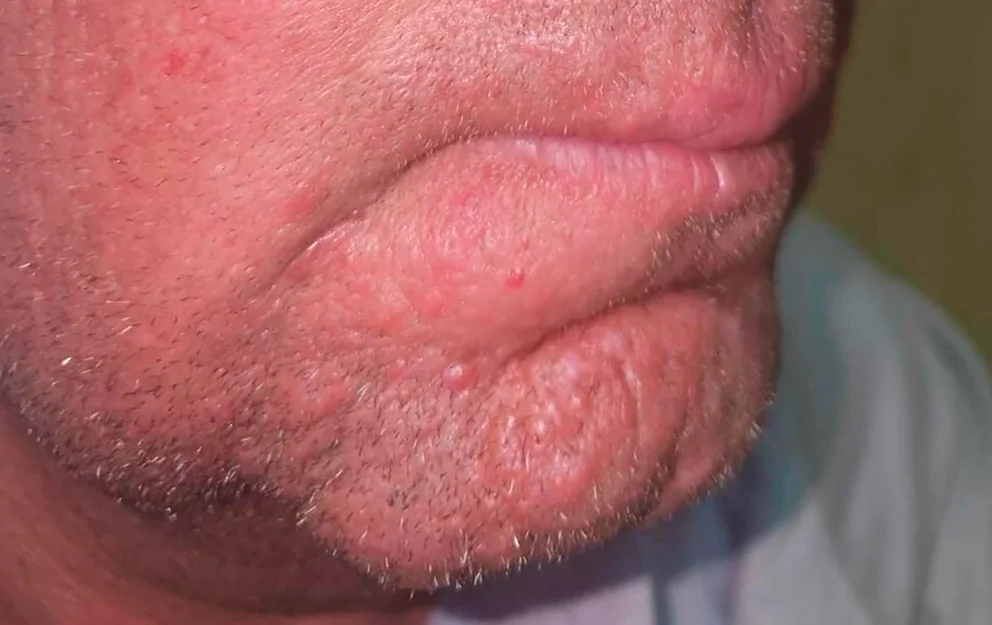
Result 2 months after the second session of CO_2_ laser treatment.

We reported herein a case of gnatophyma, an unusual form of rosacea, treated successfully with ablative CO_2_ laser. In fact, Gnatophyma, a rare variant of phyma, is characterized by an enlarged chin with hypertrophied tissue displaying irregular surface nodularities.^1^ These facial deformities significantly contribute to aesthetic discomfort. Treatment is a real challenge for dermatologists in clinical practice. Medical treatments such as oral isotretinoin and antibiotics did not treat gnatophyma; however, they aid to reduce sebum production as well as sebaceous gland size. In phyma, surgical is the first‐line treatment, including electrosurgery, dermabrasion, and ablative and nonablative laser therapy.

Due to its rarity, the management of gnatophyma is still challenging. In fact, only seven cases of gnatophyma have been documented in the literature.^1^, ^2^, ^3^, ^4^, ^5^, ^6^ Among them, only one case was managed with CO_2_ laser ablation.^2^ In this case, the patient underwent a combination therapy involving CO_2_ laser ablation alongside wire loop electrocautery, resulting in a favorable outcome. However, the details of the procedure were not specified. Conversely, the literature on CO_2_ laser treatment for rhinophyma is more extensive, with the largest study encompassing 124 patients treated in a single session with favorable responses.^7^

Treating gnatophyma with CO_2_ laser ablation offers the advantage of minimal risk of complications due to the absence of complex anatomical structures. Furthermore, CO_2_ lasers have the benefit of easy treatment and the advantage of a bloodless operating field, allowing precise control of tissue ablation to minimize complications.^7^, ^8^ However, its utilization necessitates specialized training and precautions within the operating room. Challenge also arises in the absence of a well‐defined endpoint, as observed in cases of rhinophyma where there is a clear expression of glandular content.^7^, ^9^ Thus, we exercised caution during the ablative treatment, deliberately avoiding over‐treatment to mitigate potential complications.

In summary, although treatment of this disfiguring is mandatory since its psychological impact, the diagnosis, and the management of gnatophyma may be challenging and complex for practitioners. Our case suggests the effectiveness of ablative CO_2_ laser for gnatophyma. Further studies are required to confirm our results.

## AUTHOR CONTRIBUTIONS

Amal Chamli and Houda Hammami. wrote the manuscript with support from Houda Hammami., Refka Frioui, and. Anissa Zaouak. Samy Fenniche. supervised the project. All authors have read and approved the final manuscript.

## FUNDING INFORMATION

None.

## CONFLICT OF INTEREST STATEMENT

None.

## CONSENT

The examination of the patient was conducted according to the principles of the Declaration of Helsinki. The authors certify that they have obtained all appropriate patient consent forms, in which the patient gave his consent for images and other clinical information to be included in the journal. The patient understands that his name and initial will not be published and due effort will be made to conceal his identity, but that anonymity cannot be guaranteed.

## ETHICS STATEMENT

Informed consent Written informed consent was obtained from the patient to publish this reportin accordance with the journal’s patient consent policy.

## Data Availability

Data sharing is not applicable to this article as no datasets were generated or analyzed during the current study.
